# Elastic Properties of Poly(ethylene glycol) Nanomembranes and Respective Implications

**DOI:** 10.3390/membranes12050509

**Published:** 2022-05-10

**Authors:** Zhiyong Zhao, Michael Zharnikov

**Affiliations:** Angewandte Physikalische Chemie, Universität Heidelberg, Im Neuenheimer Feld 253, 69120 Heidelberg, Germany; wu182@uni-heidelberg.de

**Keywords:** poly(ethylene glycol), STAR-PEGs, free-standing membranes, bulge test, Young’s modulus, electron irradiation, UV light, “hot” electrons

## Abstract

Free-standing poly(ethylene glycol) (PEG) membranes were prepared from amine- and epoxy-terminated four-arm STAR-PEG precursors in a thickness range of 40–320 nm. The membranes feature high stability and an extreme elasticity, as emphasized by the very low values of Young’s modulus, varying from 2.08 MPa to 2.6 MPa over the studied thickness range. The extreme elasticity of the membranes stems from the elastomer-like character of the PEG network, consisting of the STAR-PEG cores interconnected by crosslinked PEG chains. This elasticity is only slightly affected by a moderate reduction in the interconnections at a deviation from the standard 1:1 composition of the precursors. However, both the elasticity and stability of the membranes are strongly deteriorated by a strong distortion of the network imposed by electron irradiation of the membranes. In contrast, exposure of the membranes to ultraviolet (UV) light (254 nm) does not affect their elastic properties, supporting the assumption that the only effect of such treatment is the decomposition of the PEG material with subsequent desorption of the released fragments. An analysis of the data allowed for the exclusion of so called “hot electrons” as a possible mechanism behind the modification of the PEG membranes by UV light.

## 1. Introduction

Poly(ethylene glycol) (PEG) is a material that has distinct bioinert properties. It is frequently used for the suppression of adhesion and settlement of biomolecules and bio-organisms [1,2,3,4,5,6] as well as for the fabrication of biologically inert templates that can be decorated with bioactive functional groups and specific receptors [7,8,9]. For these applications, PEG moieties are used either as terminal parts of self-assembled monolayers (SAMs) or as the major or even only component of thin films. Such films, in particular, can be prepared by spin-casting, drop-casting or doctor blading of acrylate- or isocyanate-decorated PEGs, dissolved in a suitable solvent, with the subsequent crosslinking of individual precursors. The latter process is mediated by either photochemical reactions triggered by exposure of the films to ultraviolet (UV) light [10,11,12] or by chemical coupling [13,14], which relies on the mutual affinity of specific coupling groups embedded into the PEG precursors. As such precursors, both linear and branched PEGs can be used, with the latter moieties being more suitable for efficient crosslinking. Usually these moieties, termed as STAR-PEGs, are comprised of several PEG arms coupled together in the joint center and decorated with terminal groups, which are responsible for crosslinking or serving as specific receptors. Particularly useful terminal groups in this context are amine and epoxy, having complementary affinity and reacting by the formation of ethanol-amine-like bonds. By decorating individual STAR-PEG precursors with these groups, efficient crosslinking can thus be performed, resulting in a stable all-PEG network. This strategy has indeed been realized using amine- and epoxy-substituted, four-arm STAR-PEGs. Thermoactivated crosslinking of the respective STAR-NH_2_ and STAR-EPX precursors allows for the fabrication of exceptionally stable ultrathin PEG films and coatings [15,16]. These films exhibit pronounced bioinert and hydrogel properties and their characteristics could be flexibly tuned by varying the parameters of the preparation procedure [15] and the molecular weight of the precursors [16]. Further options are provided by the creation of hybrid materials on the basis of these films [15], their decoration with bioreceptors [8], and their modification by electron irradiation and UV light [17,18], paving the way to a variety of lithographic applications.

In addition, PEG films can be separated from the substrate [19] and subsequently transferred to a secondary substrate in the context of a specific application [20,21] or even exist as a free-standing membrane [16,19]. These membranes are quite stable and possess an exceptional elasticity, emphasized by a very small Young’s modulus of 2.1–5.2 MPa at a thickness of ~100 nm [16]. The value of Young’s modulus depends on the molecular weight of the STAR-NH_2_ and STAR-EPX precursors [16], but is most likely a function of the membrane thickness and its composition at a deviation from the standard 1:1 ratio between STAR-NH_2_ and STAR-EPX, which is useful for some applications [8]. It cannot also be excluded that the derived values of Young’s modulus are affected by the parameters of the experimental setup used for their measurement, which will strongly diminish their reliability.

These issues are specifically addressed in the present study in which we also explore the possibility of tuning the elastic properties of the PEG membranes by the modification of the parent films by electron irradiation and UV light. The latter experiments, in combination with additional measurements, also shed some light onto the effect of UV light on PEG materials, which is still controversially discussed in the community [18].

## 2. Materials and Methods

**Figure 1:**
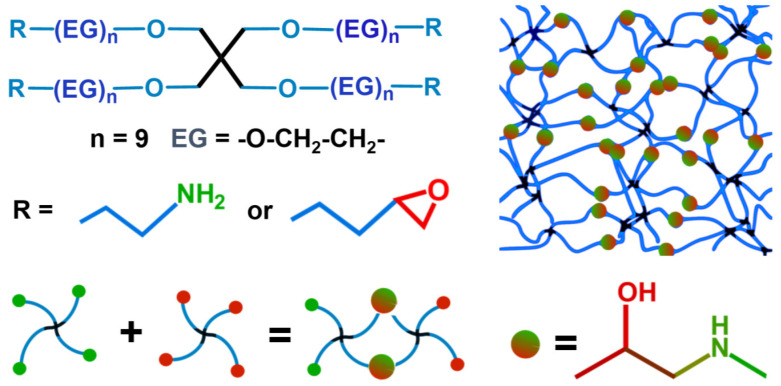
The structure of the STAR-NH_2_ and STAR-EPX precursors and a schematic drawing of the reaction between the terminal epoxy and amine groups of the precursors resulting in the appearance of ethanol-amine-like crosslinking bonds. This reaction mediates the formation of porous PEG film, which can then be separated from the substrate as a free-standing membrane.

**Chemicals.** STAR-NH_2_ and STAR-EPX compounds (Figure 1) were purchased from Creative PEGWorks (USA) and used as received. The molecular weight (MW) of these compounds was 2000 g/mol, so that the PEG arms comprised 9–11 EG monomers and had a length of 3.5–3.9 nm. The compounds were characterized by low polydispersity and high purity, viz. 99% for STAR-NH_2_ and 98% for STAR-EPX in terms of amine and epoxy substitution, respectively.

**Membranes Fabrication.** PEG membranes were prepared according to the established literature procedure [15,16] as schematically illustrated by Figure 1. As the first step, STAR-NH_2_ and STAR-EPX compounds were individually dissolved in chloroform with the same concentration, mixed together, spin-coated onto the substrate (SiO_2_-passivated Si wafers), and crosslinked by thermal annealing (6 h, 80 °C). According to the previous data [15], viz. the evolution of the characteristic vibration of the epoxy group in infrared reflection absorption spectra, the degree of conversation for the crosslinking reaction was ca. 97% for the given conditions and a mixing ratio of 1:1 (wt./wt.), which was selected in most of the experiments. However, this ratio was varied to some extent in the dedicated experiments (see Section 3.3). As the second step, the PEG films were extensively rinsed with ethanol to remove possible weakly bound material. Subsequently, the resulting PEG films on SiO_2_-passivated Si wafers were exposed to HF to diminish their bonding to the substrate by the removal of the SiO_2_ overlayer, followed by the separation from the substrate using an oblique immersion in water. Finally, a secondary substrate was put below the floating PEG nanomembrane, which was then lifted from the water surface and carefully dried with water-absorbing paper. As secondary substrates, we used custom-fabricated metal frames with a circular window, suitable for bulge test (see below). For some dedicated experiments, PEG films were prepared on gold substrates (100 nm Au on Si) following the same procedure as described above. Separation of the films from the substrates was performed according to the literature procedure [19].

**Optional Modification of Membranes.** The membranes were studied either as prepared or after being additionally treated by electron irradiation or exposed to UV light before their separation from the primary substrate. The electron irradiation was homogenously applied using a flood gun (FG20, Specs, Berlin, Germany). The treatment was performed at room temperature and under ultrahigh vacuum (UHV) conditions with a base pressure of ca. 1 × 10^−8^ Torr. The energy of electrons was set to 50 eV. The electron flux was monitored by a Faraday cup.

The UV irradiation was homogeneously applied as well. The films were exposed to UV light with a wavelength of 254 nm provided by a short-wave (UV-C) Hg-vapor lamp (Benda Konrad Laborgeräte, Wiesloch, Germany). The treatment was performed at ambient conditions and room temperature. The distance between the UV source and samples was ~2.5 cm. The intensity of the UV light was set to 2 mW/cm^2^ and monitored by a suitable UVX radiometer sensor (Ultra-Violet Products Ltd., Upland, CA, USA). After the irradiation, the samples were washed with the solvent to remove weakly-bound material.

**Elastic Properties.** The stability and elastic properties of the PEG membranes were monitored by the bulge test, which is a well-established and reliable technique for this purpose [22,23,24]. The precision of the measurements is generally better than 5%, according to literature [22]. For our experiments, we used custom-fabricated supporting frames with a circular window. The membranes were suspended over the window. The diameter of the window was 1 mm for most of the experiments but varied for some specific experiments (0.3 and 0.5 mm). The frame was glued to the end of a metal hollow cylinder and connected to a piston, allowing for the application of an adjustable pressure to the membrane. The pressure was measured by a differential-pressure transducer (SDP2000, Sensirion, Stäfa, Switzerland) while the deflection of the membrane was monitored by an optical microscope (Olympus BH-2, Olympus Corporation, Tokyo, Japan) and recorded using a video camera (DMK 37AUX178, Imaginesource, Bremen, Germany). Thus, deflection–expansion (load–unload) curves could be measured.

**Membrane Thickness.** The thickness of the PEG membranes was monitored by ellipsometry before their separation from the substrate. The measurements were performed with a spectroscopic ellipsometer (M-44, J.A. Woollam, Lincoln, NE, USA) at a fixed angle of incidence/reflection of 75°. The thickness was calculated by adapting the experimental data to a bilayer model consisting of an SiO_2_ layer and PEG film. The optical constants of SiO_2_ were determined using an SiO_2_/Si substrate; those of the PEG films were obtained using a Cauchy layer-dispersion relation including the first two terms and adapting both coefficients to the ellipsometric measurements.

**X-ray Photoelectron Spectroscopy (XPS).** In a few selected cases, complementary XPS measurements were performed. The measurements were carried out with a MAX 200 spectrometer (Leybold-Heraeus, Köln, Germany) equipped with an Mg Kα X-ray source (260 W; ca. 1.5 cm distance to the samples) and a hemispherical analyzer (EA 200; Leybold-Heraeus). The spectra were measured in normal emission geometry with an energy resolution of ~0.9 eV. The binding-energy scale of the spectra was referenced to the Au 4f_7/2_ emission at 84.0 eV [25].

## 3. Results and Discussion

### 3.1. Parameters of the Bulge Test

**Figure 2:**
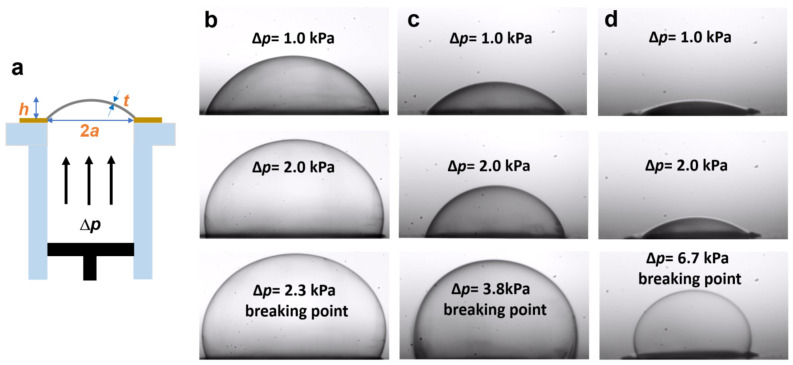
(**a**) Schematic illustration of the bulge test setup along with the relevant parameters (see text for description). (**b**–**d**) Selected optical images of the deflected PEG membranes, suspended over a circular window with a diameter of 1 mm (**b**), 0.5 mm (**c**), and 0.3 mm (**d**). The bottom image in each series corresponds to the pressure close to the breakdown point.

In the bulge test, a free-standing thin film or a membrane is suspended over a window and uniform pressure is applied to one side of the window, causing the film/membrane to deflect outwards (Figure 2a). The stress and strain in the film can then be determined from measurements of the pressure difference on both sides of the membrane (∆*p*) and the window’s deflection (*h*). The further relevant parameters are the geometrical shape and size of the window and the film thickness (*t*). In our case, a circular window was used and its diameter (2*a*; *a*—radius) was varied at 0.3 mm, 0.5 mm, and 1 mm. The membrane thickness was set to 100 nm.

Representative microscopy images of the deflected PEG membranes for these windows are shown in Figure 2b–d, respectively. At a fixed ∆*p*, the deflection strongly depends on the diameter of the window, being the largest for 1 mm diameter and progressively smaller for the smaller windows. The maximal ∆*p* corresponding to the rupture of the membranes showed an inverse behavior, being the lowest for 1 mm diameter and progressively higher for the smaller windows.

**Figure 3:**
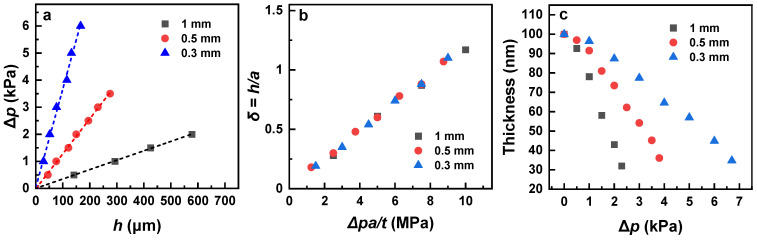
∆*p* vs. *h* (**a**) and *h*/*a* vs. ∆*p*·*a/t* (**b**) plots for the PEG membranes suspended over the circular windows with the different diameters (see the legends); straight lines in (**a**) are tentatively drawn through the experimental points. (**c**) Thickness of the membranes as a function of ∆*p*.

The entire bulk of the deflection data is presented in Figure 3a, in deflection versus ∆*p* fashion. Generally, for the bulge test, the relation between the pressure difference and the deflection of the suspended membrane over a circular window is given by [26].

(1) $$∆p=4\frac{t}{a^{2}}\sigma_{0}h+\frac{8}{3}\cdot \frac{t}{a^{4}}\frac{1}{1-v}{\mathrm{Eh}}^{3}$$

where *σ*_0_ is the residual stress, *v* is Poisson’s ratio, and *E* is Young’s modulus (*E*). Significantly, the ∆*p* vs. *h* plots in Figure 3a exhibit a nearly linear behavior for all *a* values, corresponding to the first term on the right side of Equation (1) and framing the second term as only contributing to a small extent, which means that the *E* values for the PEG membranes are very small. Further, to avoid the influence of different geometrical parameters (*a*, *t* and *h*) on the apparent sensitivity of different membranes, a dimensionless aspect ratio of deflection (*h*) to window radius (*a*), denoted as *δ*, can be plotted against ∆*p* normalized by the aspect ratio of the window diameter and membrane thickness according to the equation derived from Equation (1) (*v* was tentatively set to 0.25 [16]).

∆*p·a/t* = 4 *σ*_0_·δ *+* 3.6·*E*·*δ*^3^ (2)

The respective data are presented in Figure 3b, with the different *a* values perfectly matching each other and building together a nearly straight line, which once again indicates that the *E* values for the PEG membranes are very small. These values can be directly calculated on the basis of the strain (*σ*) and the stress (ε), which can be expressed as

*σ* = *p·a*^2^/4*th* (3)

and

ε = 2*h*^2^/3*a*^2^ (4)

Young’s modulus can then be calculated according to the equation

*E* = *σ*/ε = 3*pa*^4^/8*th*^3^ (5)

**Figure 4:**
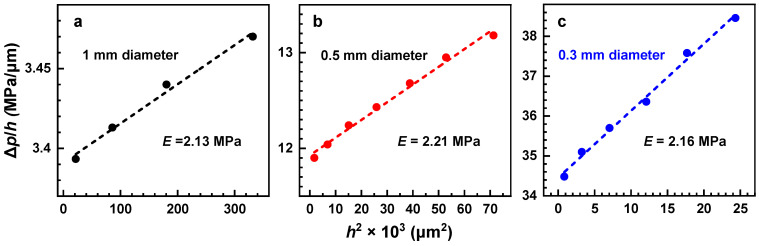
∆*p*/*h* vs. *h*^2^ plots for the PEG membranes suspended over the circular window with a diameter of 1 mm (**a**), 0.5 mm (**b**), and 0.3 mm (**c**). The straight lines are linear fits to the experimental points. The *E* values were derived from the slopes of these lines, according to Equation (1).

The calculated values for 0.3 mm, 0.5 mm, and 1 mm diameters are 2.45 MPa, 2.47 MPa, and 2.41 MPa, respectively. These values are nearly identical, which suggests that the size of the window in the bulge test experiments does not influence the result. These values agree well with the previous results for MW = 2000 g/mol, *t* = 100 nm, and *a* = 0.5 mm, viz. ~2.4 MPa, which were calculated using the same method [16]. Note also that the Young’s modulus values can also be calculated by an alternative approach, relying on the ∆*p/h* vs. *h*^2^ plots according to Equation (1) (Figure 4). The respective values of 2.13–2.21 MPa are very close to those obtained by the strain/stress method; however, they depend on Poisson’s ratio, the exact value of which for the PEG membranes is not known. Consequently, we consider evaluation of Young’s modulus on the basis of the strain and stress values as preferable.

A further interesting point is the thickness of the PEG membranes upon their stretching, which was estimated from the geometrical considerations. The respective data are shown in Figure 3c. Accordingly, the thickness changes with different rates, depending on the size of the window. However, the ultimate thickness at the stretching close to the breakdown does not vary much, and was estimated at ~32 nm, ~36 nm, and ~34.5 nm for a window diameter of 1 mm, 0.5 mm, and 0.3 mm, respectively. This correlation underlines, once again, a consistency of the bulge test data for different sizes of the window.

A final aspect is the value of the residual stress (*σ*_0_), which is a stress that remains in a material after the original cause of the stresses has been removed. The value of *σ*_0_ can either be determined from the linear fit of the ∆*p* vs. *h* plots (Figure 3a), by neglecting the second term on the right side of Equation (1), or from the ∆*p*/*h* vs. *h*^2^ plots (Figure 4), as the intersection of the linear fit with the Y-axis. The first procedure gives 185 kPa, 194 kPa, and 190 kPa for the 0.3 mm, 0.5 mm, and 1 mm windows, respectively. The second procedure gives closer values of 204 kPa, 202 kPa, and 217 kPa, respectively. In both cases, the values do not noticeably vary with the window size.

### 3.2. Effect of Membrane Thickness

**Figure 5:**
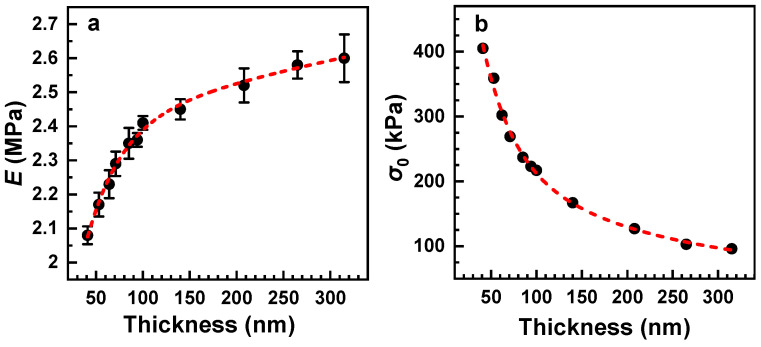
Young’s modulus (**a**) and residual stress (**b**) as functions of the membrane thickness. The experimental points are tentatively traced by the red dashed curves. The data for the thicknesses below and above 60 nm were obtained with the 0.5 mm and 1 mm windows, respectively.

The dependence of the elastic properties on the thickness of the PEG membranes was studied in the 40–320 nm range. The results for two different windows were combined together since the thinnest membranes could not be suspended over the 1 mm window without a rupture. The derived values of Young’s modulus are shown in Figure 5a. The data for the different windows perfectly match and complement each other, which is further evidence that the size of the window in the bulge test experiments does not influence the result. According to these data, Young’s modulus progressively increases with increasing film thickness, with a larger rate at small thicknesses and a lower rate at large thicknesses. Such a behavior is understandable since a thin film is easier to deform, and the effect should most likely be stronger at small thicknesses, for which the relative thickness changes more rapidly in response to a variation in the absolute thickness.

Note that the derived Young’s modulus values of the PEG membranes in the entire studied thickness range are very small, which renders these membranes extremely elastic. For comparison, the Young’s modulus of a 55 nm polyethylene membrane was estimated at 10 GPa [27], that of carbon nanomembranes of 1 nm thickness was reported to be 45 GPa [28], that of a vapor-deposited, 30 nm tris(8-hydroxyquinoline) aluminum film was 2.81–3.88 GPa [29], and that of 320 nm Si membrane was 220 GPa [30]. One of the lowest Young’s modulus values (32 MPa) was reported for ~100 nm plasma-polymerized allylamine films [31], but even this value is more than an order of magnitude higher than that for the PEG membranes.

Along with Young’s moduli, residual-stress values were calculated as well (Figure 5b). This parameter exhibits a progressive decrease with increasing film thickness. The behavior is nearly inverse to that of Young’s modulus but the relative extent of the *σ*_0_ variation is much larger.

### 3.3. Effect of Membrane Composition

**Figure 6:**
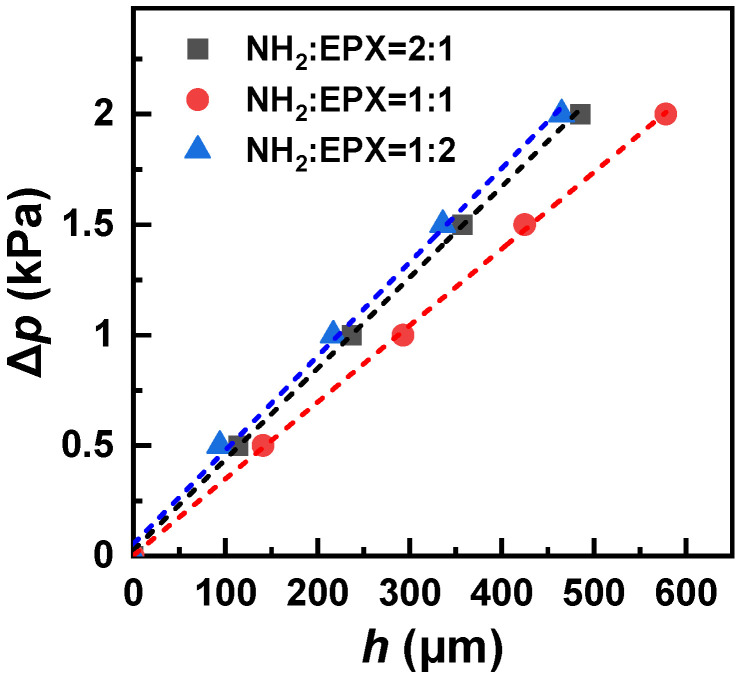
∆*p* vs. *h* plots for the PEG membranes prepared at the different mixing ratios of the STAR-NH_2_ and STAR-EPX precursors (see the legend); a nearly linear dependence is tentatively traced by the straight lines. The thicknesses of the membranes were close to 100 nm. The window diameter was 1 mm.

Generally, STAR-NH_2_ and STAR-EPX precursors should be a 1:1 mixture in order to have an optimal mixing ratio for efficient crosslinking. However, in this case, the vast majority of the amine and epoxy groups form ethanol-amine-like bridges (Figure 1) and the amount of the non-reacted groups is very small (see Section 2 for details). This situation is of advantage for most applications but is unfavorable in the case of the post-functionalization of PEG films and membranes, which relies on the reaction of a specific functional group or a receptor with the non-reacted amine or epoxy moieties. A possible solution is then a deviation from the standard mixing ratio, thereby rendering a certain amount of amine or epoxy groups free and capable of further reactions [8]. At the same time, such a deviation can change the other parameters of the films and membranes, including their elasticity. In this context, we studied the respective effect varying the STAR-NH_2_/STAR-EPX ratio to some extent, viz. as 1:2 and 2:1, and comparing the results with the reference membrane with the standard, most optimal composition (1:1). The respective ∆*p* vs. *h* plots are shown in Figure 6. For all three compositions, the experimental points exhibit nearly linear behavior, but the slope of the straight lines tracing this behavior for both of the non-optimal compositions (1:2 and 2:1) is different from that of the optimal composition. This suggests different residual-stress values in these films (see Equation (1)), which is most likely accompanied by different Young’s moduli. Indeed, the *E* values, calculated from the strain/stress relation (Equation (5)), are 4.11 MPa for the 2:1 ratio and 4.67 MPa for the 1:2 ratio, differing from the reference value of 2.41 MPa for the 1:1 ratio. Interestingly, nearly the same increase in *E* occurs due to the excess of both STAR-NH_2_ and STAR-EPX. This makes sense since a distortion of the PEG network should be similar in both cases, with some of the PEG arms not participating in the crosslinking but staying loose. These arms cannot then participate in the stress-induced stretching of the matrix, which reduces its overall elasticity.

### 3.4. Effect of Electron Irradiation

**Figure 7:**
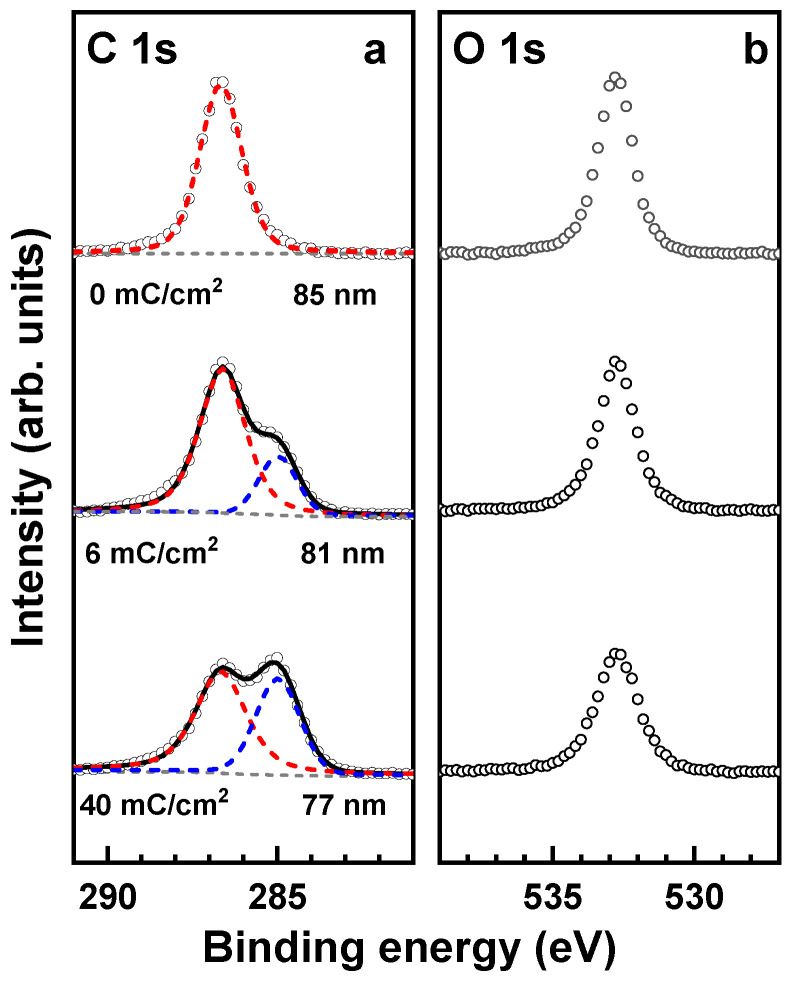
C 1s (**a**) and O 1s (**b**) XP spectra of the pristine and irradiated PEG films (open circles). The dose and thickness are given in the respective spectra. The C 1s spectra are decomposed into the components related to the pristine (red dashed line) and modified (blue dashed line) ether groups in the PEG film. The sum of these components for the irradiated films is drawn by the black solid line.

Exposure of the PEG films to electrons results in partial desorption of the PEG material and transformation of at least a part of the residual film into a carbon-enriched and oxygen-depleted matrix [17]. The respective changes, following first-order kinetics [17], can be readily monitored by XPS. The XP spectra of the pristine PEG membrane in Figure 7 show the characteristic C 1s and O 1s peaks of the intact PEG moieties at binding energies (BEs) of 286.6 eV and 532.8 eV, respectively (Figure 1). The intensity of both these peaks progressively decreases in the course of electron irradiation, and a new peak at a BE of 284.9 eV, which is characteristic of the carbon-enriched and oxygen-depleted residual matrix, appears and increases in intensity. The depletion of oxygen is additionally emphasized by the intensity ratio of the overall C 1s and O 1s signals. If we set this ratio to 1.0 for the pristine film, the values for the irradiated film will be 1.46 (6 mC/cm^2^) and 1.81 (40 mC/cm^2^). The partial desorption of the PEG material is emphasized by the thickness reduction, which decreases from 86 nm to 81 nm (6 mC/cm^2^) and further to 77 nm (40 mC/cm^2^).

Note that XPS only probes the topmost part of the PEG films, within the effective sampling depth, which is generally given by 3λ, where λ is the attenuation length of the photoelectrons depending on their kinetic energy [32]. For the PEG films and given excitation energy (see Section 2), the latter parameter was estimated at 3.9 nm (C 1s) and 3.3 nm (O 1s) [15], which gives the sampling depth of 10–12 nm. This is noticeably less than the thickness of the PEG films, so it is not clear whether the electron-induced modification encloses the entire film or only a part of it. In any case, the extent of modification is significant, which can be reflected in the elastic properties of the respective membranes.

**Figure 8:**
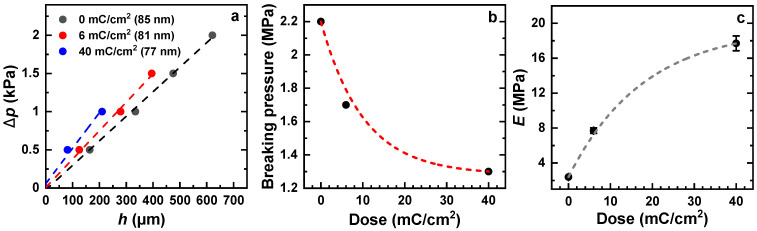
(**a**) ∆*p* vs. *h* plots for the pristine and irradiated (electrons) PEG membranes (see the legend for the thickness value); a nearly linear dependence is tentatively traced by the straight lines. (**b**) The pressure corresponding to the breaking of these membranes as a function of irradiation dose. (**c**) Young’s modulus of these membranes as a function of irradiation dose. The window diameter was 1 mm.

These properties are illustrated in Figure 8. The ∆*p* vs. *h* plots for the pristine and irradiated membranes are presented in Figure 8a. For all three samples, the experimental points exhibit nearly linear behavior, but the slope of the straight lines tracing this behavior varies with the dose, suggesting different residual-stress values. Another parameter, which varies significantly over the series, is the maximal applied pressure before the membrane breaking. As shown in Figure 8b, this pressure noticeably decreases with the irradiation dose, resulting in the progressive diminishment of the membrane stability. The Young’s moduli, calculated from the strain/stress relation (Equation (5)), show a strong dependence on the dose as well (Figure 8c), with an increase by a factor of ~3.2 at 6 mC/cm^2^ and by a factor of 7.4 at 40 mC/cm^2^. This suggests a tremendous loss in elasticity upon electron irradiation.

However, this loss is understandable, assuming that the elastomer-like PEG network (Figure 1) transforms into an oxygen-depleted carbonaceous matrix with extensively cleaved PEG arms that cannot be overstretched any more. In addition, the residuals of these arms are capable of creating additional crosslinks in the network, further limiting its elasticity. Such a tentative mechanism behind the decrease in elasticity agrees well with the earlier reported progressive loss of hydrogel properties of the PEG films upon electron irradiation [17]. Note also that electron-irradiation-induced crosslinking of aliphatic molecular assemblies is a well-known phenomenon, occurring complementary to the breaking of bonds [33,34].

### 3.5. Effect of UV Light

In contrast to electron irradiation, the exposure of the PEG films to UV light does not result in their modification but only in a partial loss of material [18]. However, the mechanism behind the respective decomposition of the PEG network, which follows zero-order kinetics [18], is still unclear. Interestingly, the exposure of closely related, PEG-substituted self-assembled monolayers (SAMs) to UV light results in the same effect as their exposure to electrons, viz. a decomposition of the PEG moieties and their chemical modification, accompanied by a depletion of oxygen [35,36,37]. The most likely mechanism behind this behavior in the UV case is the effect of so-called “hot” electrons originating from the substrate. Initially, no photoelectrons and secondary electrons from the substrate can reach molecular adsorbates since their energy at the given wavelength of the UV light is lower than the work function of the substrate. However, these electrons, termed then as “hot”, can tunnel into empty states at the substrate–adsorbate interface through the work function barrier [38,39,40,41]. Even though the penetration depth of these electrons into an organic film is limited, it is most likely sufficient to affect the entire SAM, which is just few nanometers thick.

Taking this model into account, one can reasonably assume that the modification of the PEG films by UV light is also mediated by “hot” electrons. This process will then exclusively involve the region close to the buried PEG/substrate interface, within the penetration depth of “hot” electrons, and not be traceable by any technique applied to the outer (ambient) side of the PEG film. However, the elastic properties of the respective PEG membranes should be significantly changed, since the exposure of their “bottom” side to the “hot” electrons will most likely have a similar effect on the elastic properties as that described in the previous section.

**Figure 9:**
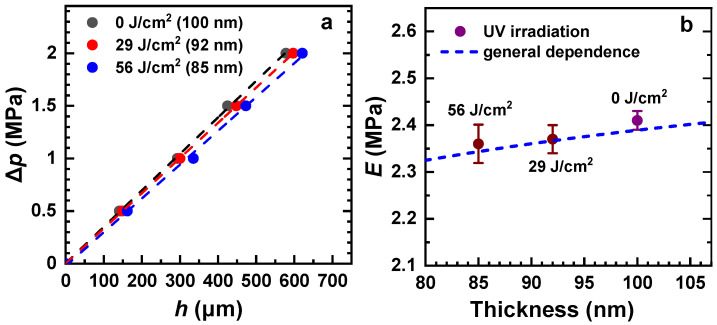
(**a**) ∆*p* vs. *h* plots for the pristine and irradiated (UV light) PEG membranes (see the legend); a nearly linear dependence is tentatively traced by the straight lines. (**b**) Young’s modulus of these membranes as a function of the membrane thickness; the UV doses are marked; the blue dashed line represents the general *E*-*t* dependence for the pristine membranes (Figure 5). The window diameter was 1 mm.

Representative ∆*p* vs. *h* plots for the pristine and exposed-to-UV-light PEG membranes are shown in Figure 9a; the decrease in the membrane thickness, given in the legend, manifests the expected effect of UV light [18]. The experimental points for all the samples lie quite close to each other, which suggests a similarity of the elastic properties. Indeed, the Young’s moduli of these membranes, calculated on the basis of the strain/stress relation (Equation (5)), do not show much variation (Figure 9b). Most importantly, in contrast to the electron-irradiation series (Figure 8c), the *E* value does not increase but decreases over the course of the irradiation treatment. Moreover, this decrease perfectly correlates with the general dependence of Young’s modulus on the membrane thickness (Figure 5), as illustrated in Figure 9b. Thus, the elastic properties of the membranes exposed to UV light are identical to those of the pristine membranes with the same thickness, which exclude their partial modification by “hot” electrons.

**Figure 10:**
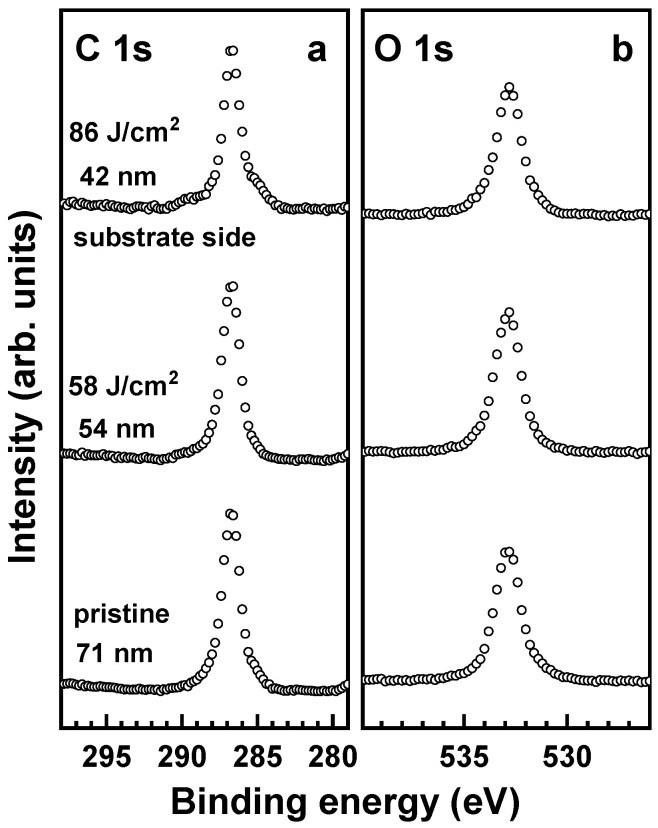
C 1s (**a**) and O 1s (**b**) XP spectra of the pristine and irradiated (UV light) PEG films deposited on Au substrate. The dose and thickness are given in the respective spectra. The bottom and middle spectracorrespond to the top side of the film facing the ambient, whereas the top spectra represent the side of the film facing the substrate. The irradiated film was turned around for the latter measurement. The bottom and middle spectra are reprinted with permission from Ref. [18]. Copyright 2021, American Chemical Society.

Further evidence for the lack of such a modification is provided by XPS, using the films and membranes on an Au substrate for direct comparison with the already reported data [18]. According to these data and as shown in Figure 10, the characteristic XP spectra of pristine and exposed-to-UV-light PEG films are nearly identical, suggesting a lack of UV-induced chemical modification. However, these spectra are representative of the topmost 10–12 nm of the PEG films and do not contain any information about possible chemical processes at the buried PEG–substrate interface. This interface could be directly accessed by the separation of the UV-treated film from the substrate and its placement onto the secondary substrate upside down, i.e., with the substrate side exposed to the XPS spectrometer. The respective spectra in Figure 10 are very similar to those of the opposite side and are distinctly different from the spectra of the films exposed to electrons (Figure 7). The low-intensity shoulders at the high BE and low BE sides of the C 1s peak in Figure 10 most likely stem from contamination on the surface of the substrate, sticking to the PEG film during its formation.

The hypothesis of “hot” electrons can thus be fully excluded as the mechanism behind the decomposition of the PEG films and membranes by UV light. However, one can ask why this effect is likely of importance for OEG-substituted SAMs and of no impact for the PEG films. A possible explanation can be the difference in the electronic coupling to the substrate for these two kinds of systems. The SAMs couple strongly to the substrate [42], relying on the chemical bond between the anchoring group of the SAM-forming molecules and the substrate [43]. Consequently, the tunneling of “hot” electrons between the respective, strongly coupled electronic systems is likely possible, as long as empty states at the molecular side of the interface are available. In contrast, the coupling between the PEG film and the substrate can in the best case be described as physisorption, so that the electronic systems are weakly coupled (if at all) and the tunneling is hardly possible. Thus, one is only left with a direct effect of UV irradiation on the PEG films and membranes promoting the fragmentation of the PEG chains in contrast to their chemical modification, such as the depletion of oxygen and chemical transformation.

## 4. Conclusions

We studied the effect of different parameters and electron/UV-light treatment on the elastic properties of ultrathin PEG membranes prepared from the amine- and epoxy-terminated four-arm STAR-PEG precursors. As the main technique, the bulge test was used. It was demonstrated that the major parameter of this approach—the size of the window—did not influence the results, which was in particular useful to monitor the dependence of the membrane Young’s modulus on their thickness. The *E* value was found to increase from 2.08 MPa to 2.6 MPa over the thickness variation of 40 to 320 nm, with a higher rate at small thicknesses. These values are very small, rendering the PEG membranes extremely elastic, due to the elastomer-like behavior of the crosslinked network. This behavior does not dramatically change but only slightly deteriorates in response to a deviation from the standard 1:1 composition of the membranes, which is characterized by a lesser extent of crosslinking. However, a significant deterioration of the elastic properties occurs upon electron irradiation of the membranes, which is associated with their extensive chemical modification, apart from a reduction in their thickness. This modification involves depletion of oxygen and changing the crosslinking pattern in the residual membrane, leading to the loss of elasticity and stability. In contrast, the exposure of PEG membranes to UV light (254 nm) only causes a reduction in their thickness and does not result in any change of their elastic properties. This is further evidence that the only effect of UV light on all-PEG films and membranes is the decomposition of the PEG material followed by desorption of the released fragments. The exact mechanism behind this behavior is not fully understood yet and should be clarified. However, one of the possible scenarios, viz. the effect of UV-induced, “hot” electrons from the substrate could be excluded by the comparison of the electron- and UV-light-treated membranes and by the additional XPS data. A most likely reason for the failure of this mechanism for the PEG membranes is their weak electronic coupling to the substrate, making the tunneling of “hot” electrons into the membrane material hardly possible.

The bioinert and ultraflexible character of the PEG nanomembranes makes them suitable for different applications ranging from extremely sensitive sensing elements in microelectromechanical systems [26,29,44,45] to biomolecule-friendly supports for biological samples in high-resolution transmission electron microscopy [19,46,47].

## Data Availability

Data presented is contained within this paper. Additional data can be provided by the authors following a request.
